# Meat Inspection Decisions Regarding Pig Carcasses Affected by Osteomyelitis at the Slaughterhouse: From Etiopathogenesis to Total Condemnation Criteria

**DOI:** 10.3390/foods13193203

**Published:** 2024-10-09

**Authors:** Melissa Alves Rodrigues, Pedro Teiga-Teixeira, Fernanda Seixas, Alexandra Esteves

**Affiliations:** 1 Independent Researcher, 5653 LL Eindhoven, The Netherlands; pedroteigateixeira@gmail.com; 2 Department of Veterinary Sciences, University of Trás-os-Montes and Alto Douro, 5001-801 Vila Real, Portugal; fseixas@utad.pt (F.S.); alexe@utad.pt (A.E.); 3 Animal and Veterinary Science Centre (CECAV), Associate Laboratory for Animal and Veterinary Sciences (AL4AnimalS), University of Trás-os-Montes and Alto Douro, 5001-801 Vila Real, Portugal

**Keywords:** osteomyelitis, meat inspection, swine, food safety

## Abstract

Osteomyelitis is a significant cause of total carcass condemnation in pigs at the slaughterhouse. The decision for total condemnation of a pig carcass for osteomyelitis is often based on traditional perceptions of the risk of pyaemia, leading to controversy among Official Veterinarians (OV) in the industry. This review aims to provide a more comprehensive understanding of the etiopathogenesis of osteomyelitis in pigs, the microorganisms involved, and the risk factors. It also highlights the urgent need for a more uniform method to evaluate osteomyelitis cases, which could significantly reduce economic losses in the industry. Lesions originating from tail-biting, tail docking, castration, teeth resection, and raw management are described as risk factors for osteomyelitis. Osteomyelitis is caused by the entry of pathogens into the animal’s bloodstream through an open wound. *Trueperella monocytogenes*, *Staphylococcus aureus*, and *Streptococcus* spp. are the most described pathogens. At slaughter, OVs condemn carcasses with osteomyelitis due to pyaemia. Signs of acute disease are essential to identify pyaemia cases. In chronic cases, total carcass condemnation can be avoided depending on the number of lesions and vertebrae affected. A clear overall image of the problem would help authorities in various countries adopt a more homogenous approach.

## 1. Introduction

Pork is the most consumed meat worldwide [1]. Its impact on the food industry and society increases the demand by consumers for safe meat [2]. The consumption of contaminated meat is still a serious food safety risk globally [3]. Meat inspection at the slaughterhouse determines if the meat is fit for human consumption [3,4]. Its importance extends to the identification, by assessment of pathological findings, of animal health and welfare problems [4,5]. Traditional post-mortem inspections consist of the incision and palpation of the carcass and organs to arrive at a meat inspection decision [4]. The inspection is focused on detecting zoonotic diseases, such as brucellosis and tuberculosis, which at present are rare to observe at the slaughterhouse [3]. Furthermore, traditional meat inspection now poses a modern food safety risk [3]. The incision and palpation of several carcasses promotes the spread of pathogens (such as *Salmonella*, *Campylobacter*, and *Yersinia*), leading to cross-contamination [3,4]. The European Union now encourages a more modern meat inspection, where an Official Veterinarian (OV) makes a sanitary decision through risk assessment, risk management, and communication [4]. This involves visual inspection and assessing food chain information [4]. 

Many findings can lead to total carcass condemnation during the post-mortem inspection of pigs at the slaughterhouse [6,7]. Pneumonia, multiple abscesses, arthritis, and osteomyelitis are all significant causes of total carcass condemnation in pigs [6,7]. Osteomyelitis has been the most impactful cause of condemnation in countries such as Portugal, leading to high economic losses [7]. Osteomyelitis is an inflammatory process caused by pathogenic bacteria that affects the bone [8,9]. Osteomyelitis may affect long bones, physis, and vertebrae, causing possible fractures and spinal cord compression consecutive to vertebral osteomyelitis [10,11]. Due to the high vascularisation of the bone marrow, osteomyelitis is associated with the risk of pyaemia [9]. In the European Union, Regulation EC 2019/627 specifies that meat derived from animals affected by a generalised disease must be declared unfit for human consumption. This includes septicaemia, pyaemia, toxaemia, and viremia [12]. Thus, if signs of pyaemia are present in pig carcasses with osteomyelitis, then the entire carcass must be condemned. 

Nevertheless, pig carcasses affected by osteomyelitis are still a problem that is up for debate between OVs. Research on the risk factors and characteristics of the disease is severely lacking [7]. Determining the chronicity of the lesion constitutes an important step for the OV’s decision, as acute lesions are more likely to relate to pyaemia. However, detecting osteomyelitis lesions and characterising them as acute or chronic remains challenging [9]. 

This shortage of information can be detrimental to the OVs’ ability to apply a uniform and correct approach to osteomyelitis cases. The criteria for meat inspection decision-making are unclear, leading to controversial discussions among OVs. Unnecessary condemnations can lead to avoidable economic losses and minor food waste. On the other hand, carcasses with pyaemia mistakenly deemed fit for human consumption can represent a serious health risk for consumers [9].

This review aims to provide more clarity to the OVs on making the correct decision based on a more in-depth knowledge of the pathogenesis, the risk factors, and microorganisms involved in osteomyelitis, combined with a summary of the various meat inspection decision options. It also aims to promote the need for food safety authorities to develop more unambiguous, homogeneous and uniform guidelines regarding decision-making regarding osteomyelitis. 

## 2. Pathogenesis of Osteomyelitis

Osteomyelitis is an inflammatory process of the bone and bone marrow that leads to bone destruction [13,14]. An accumulation of pus at the lesion location often occurs [9]. It is primarily associated with bacterial infection and can be accompanied by secondary infections [13,15]. Bacteria infect the bone through three possible routes: haematogenous, local direct extension from adjacent soft tissues, and inoculation [13,15]. According to the duration of the infection and clinical signs, osteomyelitis can be classified as acute or chronic [14]. Acute osteomyelitis is a recent lesion of days or weeks involving local inflammation and purulent debris [16]. Chronic lesions are long-standing infections often accompanied by bone destruction and sequestrum formation [14].

At the early stage, after bacterial contamination and adhesion, the response is characterised by acute inflammation and oedema [15]. Following this, purulent material can spread widely through the medullary canal, and on some occasions, it can destroy the cortical bone, burst through the periosteum, and lead to infection in the surrounding soft tissues or drain externally [13]. Bone necrosis may occur when the purulent material increases pressure in the medullary cavity and thrombosis blood vessels [13]. Necrotic debris and a mixed population of inflammatory cells may fill the marrow spaces between necrotic and partly reabsorbed bone trabeculae, and a thick layer of granulation tissue may surround larger areas of necrotic bone [15]. 

Usually, in red meat animals, osteomyelitis is secondary to trauma, such as in the case of castrated wounds and umbilical infection in neonates [13]. Iatrogenic or spontaneous inoculation of infectious agents into traumatic or surgical wounds results in osteomyelitis [17] and may develop haematogenous spread. Haematogenous osteomyelitis is reported chiefly in young animals; it is commonly found in young horses and ruminants and affects mainly the metaphyseal area of the bone due to the vascular pattern [14,15,17]. 

Vertebral osteomyelitis (VO) is relatively frequent in pigs, cattle, sheep, goats, and horses [14,15]. It leads to severe pain and neurological symptoms, such as tetraparesis [9,18]. VO is an inflammation that affects the vertebrae and involves the medullar cavity [19]. In pigs, cervical osteomyelitis may occur by direct invasion of bacteria from muscular abscesses, which may result from contaminated injections [9]. Coccygeal and sacral VOs usually result from tail biting [9,15]. Identifying two or more VOs in a pig carcass indicates a haematogenous spread of pathogenic organisms [9]. Other entry sites of infectious agents associated with VO in pigs are the umbilical vein, bite wounds, and infections after tail docking [18]. Defining the entry site in live animals remains difficult, as when clinical signs of osteomyelitis become noticeable, the primary infection has usually resolved [18].

*Actinomyces bovis* infection, a common cause of mandibular osteomyelitis in cattle, can occasionally affect pigs [15]. Osteomyelitis in the oral cavity of pigs can also occur, resulting from dental pulp infection after clipping the teeth of piglets, which exposes the dental pulp [20]. Teiga-Teixeira et al. (2024) [7] reported that 94.78% of osteomyelitis cases in the anterior region of slaughtered pigs was mandibular osteomyelitis, representing 34.28% of the Total Condemnations. In this study, teeth resection was the factor that most contributed to mandibular osteomyelitis in pigs [7]. 

## 3. Microorganisms Associated with Osteomyelitis in Pigs

Osteomyelitis seems to be caused by various pathogens. 

*Trueperella pyogenes* and *Streptococcus* spp. are described as bacteria related to pyemia cases associated with osteomyelitis [8,9]. Despite belonging to commensal microbiota, both are Gram-positive bacteria that can behave as opportunistic pathogens and lead to infections [21,22]. In large animals, including pigs, *Trueperella pyogenes* is the most common causative agent of VO [15]. This agent is an opportunistic animal pathogen mainly associated with various suppurative infections in wild and domestic animals [23]. Vieira-Pinto et al. (2020) [9] performed a microbiological analysis of samples from purulent content of VO cases (*n* = 40) found during meat inspection at the slaughterhouse. They [9] isolated *Trueperella pyogenes* from 23 samples (57.5% of the analysed samples). In this recent study, *Streptococcus* spp. was isolated from 10 samples of VO cases (25% of the analysed samples) [9].

In cases where the risk of exposure is not mitigated, both *Trueperella pyogenes* and *Streptococcus* spp. should be considered a potential occupational risk for people who work in the slaughterhouse [8,9].

*Staphylococcus aureus* is a bacterium prone to affecting bones [15]. Pigs of all ages are susceptible to this pathogen’s septicaemic form of infection [24]. Vieira-Pinto et al. (2020) [9] isolated this pathogen in 5 out of 40 purulent content samples collected from VO cases found during meat inspection (12.5% of the samples). *Staphylococcus aureus* is a Gram-positive, bio-film-forming bacterium commonly found on the skin of animals and humans and is associated with skin and wound infections, abscesses, joint infections, endocarditis, pneumonia, and bacteraemia [25,26]. Additionally, it is the seventh most major cause of foodborne outbreaks in the European Union, representing 8.41% of the outbreaks linked to meat and meat product consumption in 2022 [27]. *Staphylococcus aureus* produces various enterotoxins and may cause nausea and diarrhoea [8]. It is resistant to commonly used antibiotics [28,29]. Antimicrobial-resistant *Staphylococcus aureus*, such as Methicillin-Resistant *Staphylococcus aureus* (MRSA) and Vancomycin-resistant *Staphylococcus aureus* (VRSA), are currently a significant challenge to public health [28,29]. The abuse and misuse of antibiotics in animal husbandry and treatment of human infections are the leading causes of the emergence of MRSA in animal production environments [30]. The dissemination routes of livestock-associated MRSA to humans may include direct contact with animals, incorrect food handling, and consumption of improperly cooked meat [30]. The study of Santos et al. (2020) [31] on the occurrence of MRSA CC398 in purulent lesions of piglets and fattening pigs in Portugal concluded that domestic pigs act as a reservoir for MRSA and transmit it to humans and animals. The same study states that from all 141 purulent samples collected from pig carcasses, 44 were from osteomyelitis cases. Seven samples were *Staphylococcus aureus* positive; three were MRSA, representing 6.8% of the total osteomyelitis sampled material [31]. These results raise the concern that osteomyelitis may be associated with the transmission of resistant strains by the management and consumption of contaminated meat, but further investigations must occur.

*Actinobacillus pleuropneumonia* (APP) was once described as a cause of necrotising osteomyelitis in 8- to 12-week-old pigs [32]. A case of osteomyelitis in a vertebral fracture due to *Actinobacillus pleuropneumoniae* was documented in an 8-week-old female weaned domestic pig [18]. *Actinobacillus pleuropneumoniae* is a Gram-negative bacterium that resides primarily on the tonsils and mucous membranes of the respiratory tract and, due to stress or other external or internal factors, may lead to necrotising pneumonia, pulmonary oedema, fibrinous pleuritis, and also multifocal abscesses [33,34,35]. Although *Actinobacillus pleuropneumonia* leads to pleuropneumonia and respiratory symptoms, it has been sporadically described as a cause of swine osteomyelitis and fibrinopurulent arthritis [18,35]. Furthermore, it has already been confirmed to be related to multifocal granulomatous hepatitis, nephritis, and meningitis [35]. Teiga-Teixeira et al. (2024) [7] also linked pleurisies, usually connected with *Actinobacillus pleuropneumonia* infections [36], with a higher occurrence of osteomyelitis in swine carcasses at the abattoir.

Other reported osteomyelitis-leading pathogens are *Erysipelothrix rushiopathie*, *Pseudomonas*, *E. coli*, and *Pasteurella haemolytica* [18].

## 4. Risk Factors for Osteomyelitis in Pigs

Research on the causative factors for osteomyelitis is still very scarce [7]. Based on currently available research, it is possible to associate osteomyelitis risk factors with behavioural issues in pigs, such as tail-biting, and with management practises, namely tail docking and teeth clipping.

Tail-biting lesions have long been described as a possible predisposing factor [7,10,20]. The presence of tail lesions is common in condemned carcasses due to generalised disease at the slaughterhouse [7,37,38]. In a recent study, 34.0% of pig carcasses condemned due to purulent osteomyelitis (*n* = 108/308) presented tail-biting lesions [7]. The same study reports that severe tail-biting lesions were significantly associated with a higher occurrence of osteomyelitis [7].

Tail-biting outbreaks are characterised by the pigs’ behaviour of biting others’ tails, increasing the risk of infection and carcass condemnation [39,40]. It is often associated with reduced animal welfare and may promote the spread of disease during production [19,41]. A tail lesion is an entry site for pathogens into the animal’s bloodstream. This promotes the dissemination of infection to several body organs and tissues [7]. This results in the formation of abscesses, lung lesions, arthritis, and osteomyelitis in the carcass that are detectable during post-mortem inspection [7]. Bacterial spread to vascular loops beneath growth plate cartilage results in embolic osteomyelitis and often pyaemia [42]. Associated with osteomyelitis, suppurative lesions such as purulent foci in the kidneys, lungs, and spleen during post-mortem inspection also suggest pyaemia [9,42]. Acute pyaemia may be associated with non-capsulated purulent lesions in lymph nodes, muscles, and the spleen, hyperaemia, and haemorrhages in the liver and lungs [9,43]. 

An interesting association between swine production management and the occurrence of osteomyelitis was found. Alban et al. (2015) [44] concluded that osteomyelitis was more frequent among batches produced under organic/free-range management than in conventional indoor pig production. According to these authors [44], tail lesions were more frequent among these pigs, which may explain these results. They [44] also associated their findings with the fact that there is more accessible access to antimicrobials in conventional production than in organic/free-range production, and pigs with tail lesions are more likely to be treated sooner by veterinary practitioners [44]. 

To reduce tail-biting, pig farmers often resort to tail docking [37,41,45]. As an invasive procedure, tail docking may also play a role in infections that lead to bone infections [9]. Docking consists of amputating a part of the tail, aiming to reduce the attractiveness of the tail for biting [46]. It is usually performed by the farmer or employees without anaesthesia or analgesia, using anything from teeth clippers and scissors, to gas or electrical cautery irons [46]. However, the efficiency of tail docking is limited and does not entirely solve tail biting [41,47]. Moreover, not having hygienic tail docking procedures also represents a potential risk of infection and may lead to spinal abscesses, arthritis, and osteomyelitis [9,48]. 

Another described risk factor for osteomyelitis in pigs is teeth resection. Teiga-Teixeira et al. (2024) [7] found a significant association between osteomyelitis and carcasses with clipped teeth. This study registered that 73.4% of the carcasses condemned due to purulent osteomyelitis presented clipped teeth (*n* = 226/308), and 17.2% presented ground teeth (*n* = 53/308).

Teeth resection is a controversial invasive procedure still commonly used in piglets despite being highly discouraged in the European Union. The incisor and canine teeth of the mandible and upper jaw are clipped or ground on the first days of piglets’ lives to prevent lesions in the sows’ teats and lesions caused by littermates [7]. The advantages of teeth resection are disputable because, alongside severe pain and stress, it may lead to damage and infection of the dental pulp, gums and roots of the teeth [7]. Bacteria may also access the bloodstream and disseminate to other body parts [7,49]. 

Castration wounds, brucellosis, and atrophic/necrotic rhinitis may be linked with osteomyelitis in pigs [9,13]. 

Fertner et al. (2017) [50] described a link between herd size and the occurrence of osteomyelitis. Osteomyelitis among finishers affected by tail lesions was more likely to occur in medium-sized herds than in large ones, and some of the author’s possible explanations are the better infrastructure conceived for large herds and more experienced staff that works in these herds [50].

## 5. Osteomyelitis as a Cause of Carcass Condemnation and Decision-Making Criteria

Osteomyelitis is one of the leading causes of total carcass condemnations in several countries [6,7,9,51,52,53]. However, comparing the prevalence of osteomyelitis cases among different countries is complicated. Some recent studies on the causes of swine carcass condemnations do not even mention osteomyelitis [54,55,56,57,58]. This can be explained by national regulatory authorities using different codes to record post-mortem findings. Osteomyelitis can be registered as simply “osteomyelitis” or, in other cases, be defined as “septicaemia” or “multiple abscesses” cases in epidemiological databases and documents [59]. The latter makes it practically impossible to use these data to assess osteomyelitis, as many other factors can result in septicaemia.

Carcasses affected by osteomyelitis are considered not suitable for human consumption because, in addition to having a disgusting appearance, there is the traditional perception of pyaemia occurrence risk, as bone marrow is highly vascularised and has an important role in haematopoiesis [60].

Although some cases of osteomyelitis can relate to neurological and septicaemic clinical signs, tail-biting lesions, and abscesses [7,9], the ante-mortem inspection may not be effective in detecting suspected osteomyelitis cases [9]. Osteomyelitis cases are generally detected at post-mortem inspection. At this stage, osteomyelitis is evident as a bone abscess, a deforming inflammatory process with an accumulation of pus [7]. 

According to Ninios et al. (2014) [61], acute single osteomyelitis should lead to total condemnation as acute lesions are more associated with pyaemia before slaughter, while chronic single lesions may lead to partial condemnation. Some authors describe that the best decision is total condemnation if a fibrous capsule does not surround the osteomyelitis [43]. 

In Denmark, lesions that may reflect an acute and generalised stage of infection lead to a rejected carcass. In contrast, carcasses with chronic purulent lesions (encapsulated abscesses in bones and organs) are sent to de-boning [8]. De-boning aims to detect abscesses connected to prior septicaemia that could not be detected in the rework area [8]. The result is primarily partial condemnation [59].

However, according to Baekbo et al. (2016) [8], de-boning was unnecessary to ensure food safety. These authors [8] proceeded to carry out a bacteriological exam of samples (including from abscesses and muscle) taken from 102 finishing pig carcasses sent for de-boning due to lesions indicative of prior septicaemia. They [8] showed that 6% of the abscesses and 83% of the muscle samples were sterile or below detection level. Furthermore, the only potential foodborne pathogen they isolated was *Staphylococcus aureus*, found in 15 abscesses and one muscle sample [8].

In Portugal, where osteomyelitis is the most impactful cause for carcass condemnation, most osteomyelitis cases are not differentiated as acute and chronic. Due to the perceived risk of pyaemia and the gap in objective criteria to evaluate osteomyelitis, OVs generally decide on total condemnation of the affected carcass [9]. This lack of criteria leads to inefficient resources for correct meat inspection decisions. As such, many carcasses fit for human consumption may be incorrectly condemned.

Vieira-Pinto et al. (2020) [9] conducted a study on 40 carcasses that were totally condemned due to vertebral osteomyelitis (VO) in an undifferentiated way. The same authors [9] registered that half the sampling carcasses presented had chronic lesions. A histological analysis validated the macroscopic classification of acute or chronic osteomyelitis, revealing a significant association between macroscopic and histological classifications [9]. A microbiological analysis was aimed at detecting pyaemia cases [9]. Pyaemia cases were associated with 46.8% of the generalised cases which led to total condemnation [9]. The study found that the acute characteristic is essential in identifying pyaemia cases. Moreover, after conceiving and applying a decision-making criteria scheme based on the study findings, the authors [9] concluded that 14 out of 40 osteomyelitis cases (11 were single chronic lesions and 3 involved two chronic lesions located in different places) could have been possibly spared from total condemnation. However, further studies should be conducted to support these findings. 

According to the scheme designed by Vieira-Pinto et al. (2020) [9], the decision of total condemnation in the case of VO must be taken if: (a) there is extensive contamination with purulent exudate; (b) an acute lesion is present (even if only in one vertebra); (c) more than three purulent lesions exist in different locations (including vertebrae- and pyaemia-related lesions). According to these authors [9], in chronic cases, partial condemnation (by trimming/cutting/deboning affected areas) is suitable if, after additional post-mortem procedures, a single vertebra is affected by a chronic purulent lesion or if two chronic purulent lesions were in different locations. 

In 2022, a European survey on post-mortem inspection of finishing pigs assessed the total condemnation criteria to declare meat unfit for human consumption [60]. This study concluded that nine out of forty respondents’ answers (22.5%) selected the option that specified total condemnation in the case of osteomyelitis. In contrast, eighteen out of forty answers chose the option of total condemnation in case of other post-mortem inspection findings, such as abscesses (for 50% of these answers) and acute stage of osteomyelitis (for the other 50%). Secondary to osteomyelitis, abscesses are commonly associated with pyaemia. However, osteomyelitis may also be a unique lesion [60]. In summary, it is essential to evaluate the concomitant abscesses and their distribution, to establish the source of infection, and to assess whether an osteomyelitis lesion indicates an acute stage.

**Figure 1:**
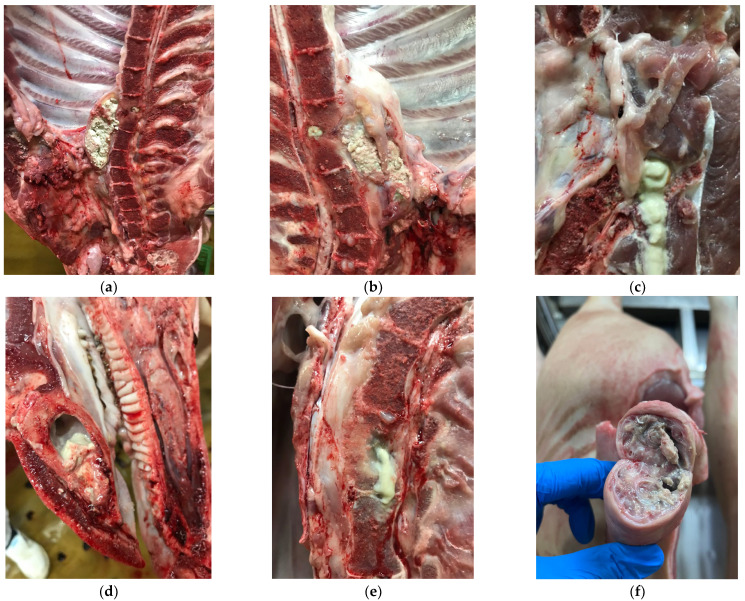
Macroscopical classification of osteomyelitis lesions in pigs’ carcasses: (**a**,**b**) are chronic osteomyelitis lesions located in the thoracic vertebrae, characterised by the presence of caseous material and a capsulated abscess at the ventral level; (**c**) acute osteomyelitis in a coccygeal vertebra, with fluid purulent exudate; (**d**) intermediate to chronic mandibular osteomyelitis presenting a more thickened pus, but still evident bone destruction and congestion; (**e**) thoracic vertebra with acute osteomyelitis with fluid pus; (**f**) chronic osteomyelitis in the caudal vertebrae associated with caseous material and tail tip necrosis.

During the post-mortem inspection procedure, osteomyelitis classification according to its chronicity may be challenging. Macroscopically, acute osteomyelitis may be represented by shiny, moist lesions with congested areas, evident bone destruction not circumscribed by adjacent remodelling tissue, and fluid purulent exudate [9]. Chronic lesions may present remodelling tissue that circumscribes moderate bone destruction without visible congested areas and thickened exudate [7]. In Figure 1, osteomyelitis lesions in the mandible and vertebrae are macroscopically classified according to the chronicity of the process.

Additional post-mortem inspection (APMI) procedures are crucial to differentiate localised and systemic cases [62]. If necessary, an incision of the iliopsoas musculature and the shoulder and topside can be performed for suspected cases of osteomyelitis and pyaemia [62]. Laukkanen-Ninios et al. (2022) [62] studied the use and variability of APMI procedures and laboratory methods as supplements for visual meat inspection of finishing pigs in Europe. They [62] found that osteomyelitis was one of the most minor conditions associated with requiring APMI procedures. According to these authors [62], thanks to the traditional splitting of carcasses, vertebral osteomyelitis is easily visible without APMI procedures. However, this statement raises a question. For market reasons, all carcasses are cut longitudinally into two portions. This permits an easy vertebral osteomyelitis assessment, but other bones are difficult to evaluate during post-mortem inspection [63]. Furthermore, the sagittal cut of the head for meat inspection is not mandatory and not applied in many (if not in the majority) of European swine slaughterhouses due to commercial reasons, and this may be a limitation in detecting mandibular osteomyelitis in pig carcasses [7]. Considering that the whole pig’s head is a piece sold, exported, and consumed in several countries, this can become an issue of quality and food safety.

## 6. Conclusions

Osteomyelitis causes considerable economic losses to the pork industry. However, studies about the disease and its assessment during post-mortem inspection at the slaughterhouse remain scarce. This condition is often caused by *Staphylococcus aureus*, *Trueperella pyogenes*, and *Streptococcus* spp. infections. Invasive farm procedures, open wounds, and compromised welfare are all risk factors for osteomyelitis. Bone infections by *Staphylococcus aureus* are considered a major food safety risk in pig carcasses. Osteomyelitis is a common cause of total carcass condemnation in pig carcasses. The most affected locations are the lumbar and thoracic vertebrae. Cases of osteomyelitis usually indicate a generalised infection in the carcass, which deems it unfit for human consumption. Recognising signs of pyaemia is vital for a correct judgement by the OV during the post-mortem inspection of pigs. However, identifying such signs is difficult, and information about their assessment is unclear. This lack of information and research leads to different opinions and decisions among OVs. 

A crucial step in the inspection process is to identify the lesion as acute or chronic. Acute osteomyelitis cases are more likely indicators of pyaemia being present in the carcass. Acute lesions contain a shiny and moist purulent exudate often associated with congestion. Chronic lesions present remodelling tissue that clearly contains the purulent exudate in the lesion. Bone destruction is moderate, and the purulent material is thick and more caseous. Congestion is rare in older osteomyelitis cases. Thus, carcasses with chronic osteomyelitis may be spared from total condemnation. However, more studies are needed to confirm this hypothesis, as clear evidence is lacking. 

More informed decision-making by OVs in osteomyelitis cases promotes uniformity and correctness in the food safety inspection of pig carcasses. The development of standard guidelines within the European community for this specific inspection finding is essential to avoid further economic losses.

Based on this review and the data currently available, the advised decision of osteomyelitis cases in finishing pig carcasses is the condemnation of the entire carcass and viscera if (1) at least one acute osteomyelitis lesion is present; (2) more than one chronic osteomyelitis lesion is found in the carcass, and (3) a chronic osteomyelitis lesion is associated with other suppurative lesions (such as multiple abscesses in the lungs) or lesions associated with pyaemia (such as renal petechiae). These conditions help prevent food waste while still promoting food safety standards.

Moreover, it is essential not to overlook findings of suppurative lesions after the post-mortem inspection that may have gone unnoticed during post-mortem examination, such as mandibular osteomyelitis in carcasses where the head is not sectioned.

The importance of good management practises to prevent or reduce the incidence of osteomyelitis is highlighted. A careful and hygienic procedure for teeth resection and tail docking is recommended, along with monitoring of tail biting lesions and investment in swine welfare measures at the farm level.

## Data Availability

No new data were created or analyzed in this study. Data sharing is not applicable to this article.
